# Systematic review of lung function and COPD with peripheral blood DNA methylation in population based studies

**DOI:** 10.1186/s12890-017-0397-3

**Published:** 2017-03-20

**Authors:** Matthew Machin, André F. S. Amaral, Matthias Wielscher, Faisal I. Rezwan, Medea Imboden, Marjo-Riitta Jarvelin, Ian M. Adcock, Nicole Probst-Hensch, John W. Holloway, Deborah L. Jarvis, on behalf of the ALEC study

**Affiliations:** 1 School of Medicine, Imperial College London, London, UK; 2 Population Health and Occupational Disease, NHLI, Imperial College London, London, UK; 3 MRC-PHE Centre for Environment and Health, Imperial College London, London, UK; 4 Department of Epidemiology and Biostatistics, School of Public Health, Imperial College London, London, UK; 5 Human Development and Health, Faculty of Medicine, University of Southampton, Southampton, UK; 6 Swiss Tropical and Public Health Institute, Basel, Switzerland; 7 University of Basel, Basel, Switzerland; 8 Airways Disease Section, NHLI, Imperial College London, London, UK

**Keywords:** COPD, Lung function, DNA methylation, Epigenetics, Peripheral blood

## Abstract

**Background:**

Epigenetic variations in peripheral blood have potential as biomarkers for disease. This systematic review assesses the association of lung function and chronic obstructive pulmonary disease (COPD) with DNA methylation profiles in peripheral blood from population-based studies.

**Methods:**

Online databases Medline, Embase, and Web of Science were searched. Google Scholar was searched to identify grey literature. After removing duplicate articles, 1155 articles were independently screened by two investigators. Peer reviewed reports on population-based studies that examined peripheral blood DNA methylation in participants with measured lung function (FEV1, FEV1/FVC ratio) or known COPD status were selected for full-text review. Six articles were suitable for inclusion. Information regarding study characteristics, designs, methodologies and conclusions was extracted. A narrative synthesis was performed based on published results.

**Results:**

Three of the six articles assessed the association of COPD with DNA methylation, and two of these also included associations with lung function. Overall, five reports examined the association of lung function with DNA methylation profiles. Five of the six articles reported ‘significant’ results. However, no consistent CpG sites were identified across studies for COPD status or lung function values.

**Conclusions:**

DNA methylation patterns in peripheral blood from individuals with reduced lung function or COPD may be different to those in people with normal lung function. However, this systematic review did not find any consistent associations of lung function or COPD with differentially methylated CpG sites. Large studies with a longitudinal design to address reverse causality may prove a more fruitful area of research.

**Trial registration:**

PROSPERO 2016: CRD42016037352.

**Electronic supplementary material:**

The online version of this article (doi:10.1186/s12890-017-0397-3) contains supplementary material, which is available to authorized users.

## Background

Epigenetics is the regulation of gene expression which is independent of the underlying DNA sequence, and is instead brought about by modifications to histones, changes in chromatin structure, microRNAs, non-coding RNAs, and DNA methylation. DNA methylation is fundamental for normal development and growth—responsible for imprinting of genes, inactivation of the X chromosome and cell differentiation [1, 2]. In humans, the majority of DNA methylation comprises the addition of a methyl group to cytosine bases within cytosine-guanine (CpG) DNA sequences [3]. These CpG base-pairs are concentrated in relatively high densities in areas known as CpG islands (CGIs) [4, 5]. DNA methylation acts to reduce the activity of transposons, such as Alu repeats, and thus contributes to genomic stability. The interaction between DNA methylation and gene expression is complex. However, it has been long theorised that DNA methylation in CGIs achieves gene repression by preventing transcription factors from binding to promoter regions and by influencing local chromatin remodelling [6].

The use of DNA methylation profiles in peripheral blood as a biomarker for risk of disease, risk of disease progression, response to therapy and as a biomarker of exposure to environmental insults that may influence disease is an attractive concept as it could be translated into clinical practice with relative ease. Recent advances in microarray-techniques have meant that epigenome-wide association studies (EWAS) are possible, although the cost of these arrays remains high, limiting application to all relevant studies.

Chronic obstructive pulmonary disease (COPD) is estimated to affect 334 million people worldwide [7, 8] with a global prevalence of 11.7%. It ranks 9^th^ in the all-cause global disability-adjusted life-years lost. The main risk factors for COPD are age and cigarette smoking [9–13], but a history of tuberculosis [14], and perhaps exposure to biomass [15] is also important in low and middle income countries. Variation in methylation in response to smoking has been reported in several CGIs [16], which may reverse with smoking cessation [17], but methylation markers for COPD and lung function have only been explored in a few studies.

This systematic review examines the association of COPD and lung function with global, epigenome-wide, and locus-specific DNA methylation in peripheral blood from population-based studies.

## Methods

The protocol was registered with PROSPERO (CRD42016037352), and methods and reporting followed the PRISMA guidelines [18].

### Search strategy

Searches comprising of 92 terms were applied to Medline, Embase and Web of Science (WoS) (see Additional file 1: Table S1) on 10 March 2016. Search terms consisted of the Medical Subject Headings (MeSH) for epigenetics, DNA methylation, COPD, cigarette smoke and lung function, in addition to other relevant keywords (e.g. hypermethylation) inputted as free text. Searches relating to lung function, COPD and cigarette exposure were carried out and combined with the “OR” Boolean operator. Searches relating to epigenetics, DNA methylation and global methylation were performed and again combined with the “OR” Boolean operator. The two searches were then combined using the “AND” Boolean operator to ensure that only studies regarding both DNA methylation and COPD or lung function were retrieved.

Searches for articles on Medline (MEDLINE In-Process & Other Non-Indexed Citations and Ovid MEDLINE) and Embase (Embase Classic + Embase) were based on “Title” and “Abstract”. The WoS platform was used to search for “Title” of articles in its Core Collection. Simplified searches consisting of topic headings for COPD, lung function and DNA methylation were carried out in Google Scholar to identify grey literature (i.e. published in non-commercial form or that falls outside the mainstream of journal and monograph publications).

Duplicates were identified and removed.

### Study inclusion

Titles and Abstracts of articles were screened by two authors (DLJ and MM) against the predefined inclusion criteria (Table 1) to identify articles for full-text review.

**Table 1:** Inclusion criteria for studies

Criteria	
1	Study included COPD patients as a comparator group or a population defined by lung function
2	Participants must be free of malignancies
3	Articles must be original research and have been published as a full-text in a peer-reviewed journal
4	Studies must have been carried out in humans (exclusion of in vivo and in vitro study designs)
5	Studies must assess relative levels of DNA methylation
6	Analysis of DNA methylation must have been in peripheral blood
7	Abstracts must be available in English.

Only articles assessing the association of lung function and COPD with DNA methylation were included for full-text review. Independent full-text reviews were performed on potentially relevant studies. The reference list of all included studies was screened to identify further studies, and citations published before the date of the main search (identified through WoS Citation Indexing tool) were examined. Articles without an abstract in English were excluded.

### Study eligibility

Articles selected for inclusion had participants with lung function measures or COPD status ascertained. COPD patients are at increased risk of lung cancer [19], but studies of COPD and lung function in lung cancer patients were excluded, as DNA methylation patterns may be influenced by lung cancer [20]. Conference abstracts were excluded. A hierarchy of exclusion was designed to categorise reasons for article exclusion during screening of titles and abstracts, but there were discrepancies in the categorisation of the reasons for exclusion of studies between investigators (most excluded articles had multiple reasons for exclusion). We did not attempt to resolve these discrepancies. However, studies which either investigator identified for full-text review were discussed, and studies were only included if both investigators agreed they were suitable for inclusion.

### Data extraction

Study identification (title, author and PubMed ID), study design (study group, control group, setting, recruitment and demographics including smoking history and lung function), methodologies (cell type, method of DNA methylation measurement, coverage, statistical analysis, correction methods), results (DNA methylation sites, β-values) and conclusions were extracted into a standardised template in Microsoft Excel 2013.

### Quality assessment

All studies were observational, had different designs, and different methodologies. The validity and bias of each study were assessed in regards to study size, study design, participant selection, adjustment for confounders, laboratory quality control, and validity of conclusions.

### Justification of narrative

Quantitative analysis was not performed. Studies were heterogeneous, and tested and reported effect estimates for different CpG sites. A narrative synthesis was performed to summarise the included articles.

## Results

### Literature search

**Fig. 1:**
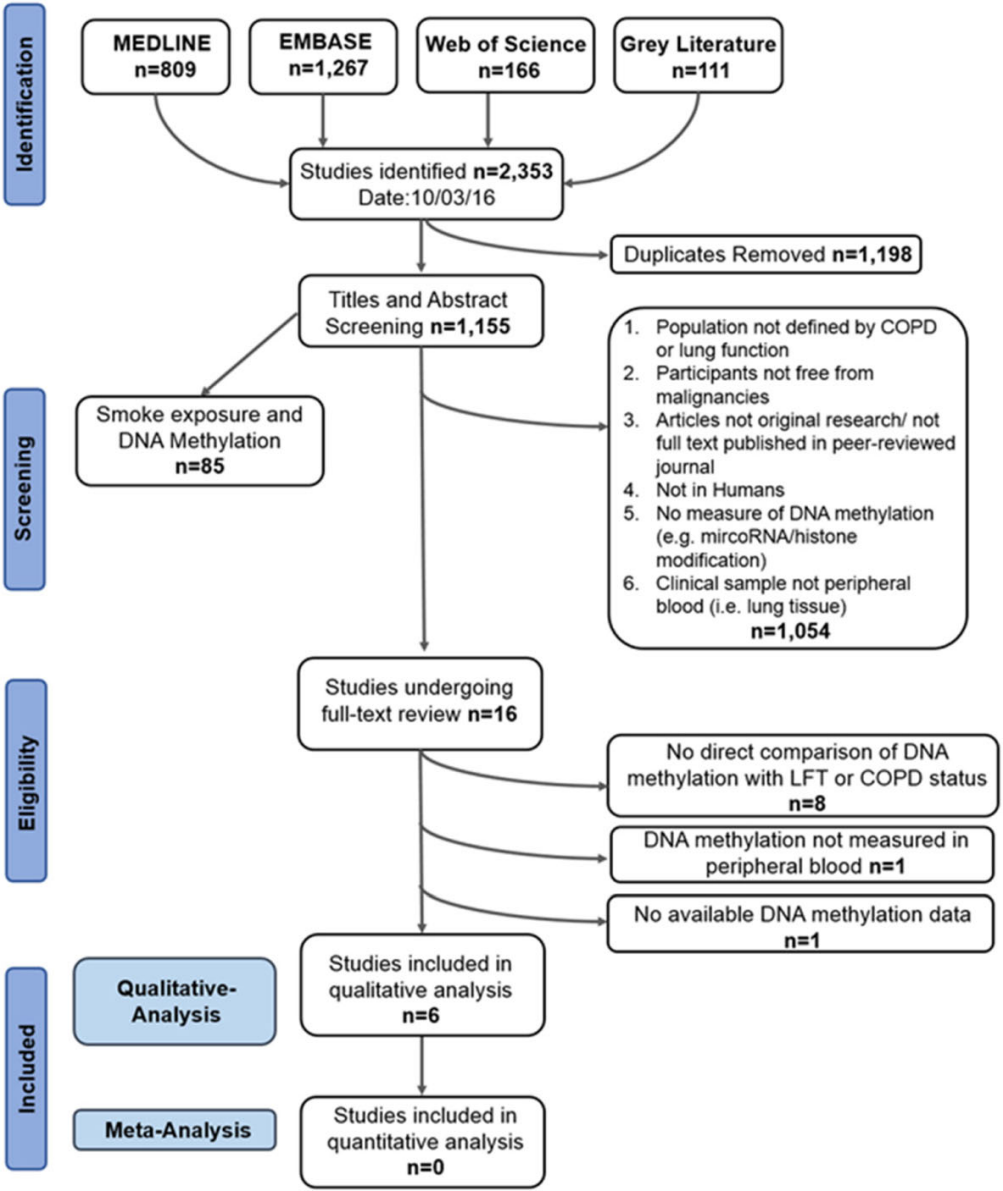
The PRISMA flow-diagram illustrating the study selection process

Searches of electronic databases identified 2242 articles (Medline: 809, Embase: 1267, and WoS: 166). Searches using Google Scholar identified 111 articles resulting in a total of 2353 potential articles (Fig. 1).

Removal of duplicates (*n* = 1198) left 1155 articles for title and abstract screening. This step excluded 1139 articles, leaving 16 articles for full-text review. Having read the full articles, six studies were considered as appropriate for systematic review.

### Study approaches

Three studies [21–23] adopted an agnostic approach performing EWAS. Two studies [24, 25] used a candidate-loci approach measuring DNA methylation at pre-determined CpG sites. One study [26] assessed global methylation across repeat elements Alu and LINE-1.

Three articles [23, 24, 26] compared DNA methylation patterns of COPD patients with healthy controls. Five studies [21–23, 25, 26] evaluated the association of lung function with DNA methylation and, of these, two (Qiu et al. and Lange et al.) also reported associations for COPD.

Underlying epidemiological designs were different. Qiu et al. [23] performed cross-sectional analyses on two family-based cohorts using the International COPD Genetics Network (ICGN) as the discovery cohort and the Boston Early Onset COPD Study (EOCOPD) as the replicative cohort. Four reports were based on cross-sectional analyses within cohort studies [21, 22, 25, 26]. Wielscher et al. [24] adopted a unique approach testing candidate CpG sites that had been identified from EWAS in lung tissue biopsies from COPD patients. The Normative Ageing Study cohort was the basis of two of the included studies [25, 26], both of which carried out cross-sectional analysis.

### Study characteristics

Tables 2 and 3 provide further details on studies and participants of the included articles. Articles were published from 2011 to 2015, and were based in the UK [21, 22], Austria [24] and USA [23, 25, 26]. The Lothian Birth Cohort 1936 [21] is the study of those born in Scotland in 1936 and continued to live in the Lothian area between 2004 and 2007 [27]. The Twins UK cohort [22] is the follow-up of adult twins (mainly, but not exclusively, female) recruited through media campaigns from the early 1990s [28]. The Normative Ageing Study [25, 26] is the follow-up of American male veterans of World War II and the Korean War. The ICGN and EOCOPD cohorts [23] comprise the follow up of patients with COPD and their families, with cases largely recruited from surgical units and pulmonary clinics. Wielscher et al. selected patients from the Medical University of Vienna between the years 2008–2012 [24].

**Table 2:** Characteristics of studies included in review

Study	Year	Location	Cohort	Population	Selection	COPD Definition
Qiu et al. [23]	2012	Multi-centre^a^	ICGN	Family-cohort of probands of COPD patients and healthy siblings/parent with >5 pack-year history of smoking	Information not available	Post-bronchodilator FEV1/FVC <0.7 and FEV1 < 70% predicted
		Boston (USA)	EOCOPD	Family-cohort of probands of COPD patients with varying severity, prior to age 53, exclusion of AAT deficiency (confirmed by serum analysis), all family members had potential to be enrolled (independent of smoking status)	Cases identified from Lung Transplant/Reduction Surgical Programs at Brigham and Women’s Hospital and Massachusetts General Hospital. Pulmonary Clinics at these hospitals and associated hospitals served as additional source of participants [32]	Post-bronchodilator FEV1/FVC < 0.7 and FEV1 < 70% predicted
Bell et al. [22]	2012	UK	Twins UK	Healthy unselected volunteers who are a twin (MZ, DZ and singleton) representing the general population	Participants recruited from media campaigns, initially only middle-aged women were included in the registry but from 1995 onwards, men and women >18 years of age were also recruited [28]	Analysed lung function only (no COPD diagnosis) – FEV1 and FVC
Lepeule et al. [25]	2012	USA	Normative Ageing	Healthy male participants at enrolment, containing smokers and ex-smokers (Veterans Administration 1963 closed-cohort)	Enrolled after an initial health screening determined that they were free of known chronic medical conditions	Analysed lung function – FEV1, FVC, MMEF (did not specify pre-/post-bronchodilator)
Lange et al. [26]	2012	Boston (USA)	Normative Ageing	Healthy male participants at enrolment, containing smokers and ex-smokers (Veterans Administration 1963 closed-cohort)	Enrolled after an initial health screening determined that they were free of known chronic medical conditions	GOLD stage II or higher (pre-bronchodilator FEV1/FVC < 0.7 and FEV1 < 80% predicted)
Marioni et al. [21]	2015	Scotland (UK)	Lothian Birth	Childhood inception cohort of ‘healthy^b^’ participants with varying lung function, containing smokers, ex-smokers and never smokers	Individuals born in 1936 in the Lothian area were identified using the Community Health Index (registered at a general practitioner) or through media advertisements. The majority of the cohort were participants in the Scottish Mental Survey 1947 [27]	Analysed lung function only (no COPD diagnosis)- FEV1
Wielscher et al. [24]	2015	Austria	N/A	COPD patients, GOLD 0 COPD patients and healthy controls	Selection of patient from Medical University of Vienna, 2008-2012	Post-bronchodilator FEV1/FVC <0.7

*Abbreviations*: *ICGN* International COPD Genetics Network

^a^Multi-centre: Cambridge, Copenhagen, Denver, Harvard, Holland, Italy, Liverpool, Nebraska, Spain, Vancouver EOCOPD: Early-onset COPD (Boston), AAT: Alpha-1-Antitrypsin, UK: United Kingdom, MZ: Monozygotic twin, DZ: Dizygotic twin, USA: United States of America, GOLD: Global Initiative for Chronic Obstructive Lung Disease

^b^Healthy: participants free from chronic disease at enrolment, GOLD stage 0: FEV1/FVC ratio > 0.7 but with respiratory symptoms

**Table 3:** Characteristics of participants in reviewed studies

Study	n	n COPD patients	n controls	Age (mean ± SD)	FEV1 (L) (mean ± SD)	FEV1/FVC% (mean ± SD)	Pack-years (mean ± SD)	%Current smoker	% Female
Qiu et al.[23]	1085	620	325	57.3 ± 8.1	N/A	54.4 ± 19.8	41.7 ± 26.2	36.5	45.6
	369	181	109	47.5 ± 7.1	N/A	54.3 ± 21.1	28.7 ± 23.6	27.9	64
Bell et al. [22]	172	N/A	N/A	57.2 ± 8.2	2.46 ± 0.46	N/A	N/A	15.1^a^	100
Lepeule et al. [25]	756	81 + 42^b^	N/A	73.3 ± 6.7	2.50 ± 0.6	N/A	20.5 ± 25.7	4	0
Lange et al. [26]	663	107	N/A	72.7 ± 6.7	2.70 ± 0.64	75 ± 8	30.6 ± 24.8	7	0
Marioni et al. [21]	1091	N/A	N/A	69.8 ± 0.78	2.36 ± 0.69	N/A	N/A	N/A	50.4
Wielscher et al. [24]	204	42	27 healthy + 34 GOLD grade 0	53.4± 13.4^c^	N/A	N/A	N/A	N/A	23.2^d^

^a^Value from entire Twins UK cohort obtained from reference [28]

^b^n COPD patients: 81 chronic bronchitis & 42 emphysema

^c^Information provided by the author Wielscher (not available in the original text)

^d^Information provided by the author Wielscher (not available in the original text)

Number of participants included in the reports ranged from 172 (Bell et al.) to 1458 (Qiu et al.). Marioni et al. also had a relatively large population (*n* = 1091) with other reports consisting of lower population numbers. The Lothian Birth Cohort 1936 cohort and the Normative Ageing Study were older than participants in other studies. COPD participants in the EOCOPD cohort (47.5 ± 7.1) were younger due to selection criteria (cohort selected for age <53).

There were broadly equal numbers of men and women in the ICGN, EOCOPD and Lothian Birth Cohort 1936 (45.6 female, 64 female and 50.4% female respectively) and a lower proportion in Wielscher et al. The Twins UK cohort used by Bell et al. was based entirely on women, whereas the Normative Aging Study was entirely male.

Definitions of COPD differed across studies.

Previous cigarette exposure, assessed as pack-years, varied widely across individuals and across studies. Reported means and standard deviations of pack-year histories from ICGN, EOCOPD and Normative Ageing Study imply highly skewed distributions. Information on previous smoke exposure was not reported for three of the studies [21, 22, 24].

Forced expiratory volume in 1 s (FEV1) was reported in four studies [21, 22, 25, 26]. Mean FEV1 values were similar across studies.

### Study methods

Tables 4 and 5 provide information on the laboratory methods for each report. Three [21–23] utilised bisulfite microarrays in EWAS. Two of these reports [22, 23] used the Illumina Infinium HumanMethylation27 BeadChip and one [21] used the Illumina Infinium HumanMethylation450 BeadChip.

**Table 4:** Laboratory methods of studies performing Epigenome-Wide Association Studies

Study	Year	Sample analysed	Methylation Analysis Technique	Coverage Level	Blood cell-type Correction
Qiu et al. [23]	2011	Leukocytes	Bisulfite Microarrays	Infinium HumanMethylation27 BeadChip	No
Bell et al. [22]	2012	Leukocytes	Bisulfite Microarrays	Infinium HumanMethylation27 BeadChip	Yes (proportion of lymphocytes)
Marioni et al. [21]	2015	Leukocytes	Bisulfite Microarrays	Infinium HumanMethylation450 BeadChip	Yes (proportion of leukocytes^a^)

^a^basophils, monocytes, lymphocytes, eosinophils and neutrophils

**Table 5:** Laboratory methods of studies performing candidate-loci or global methylation studies

Study	Year	Sample analysed	Methylation Analysis Technique	Coverage Level	Blood cell-type Correction
Lepeule et al. [25]	2012	Leukocytes	PCR and Pyrosequencing	9 candidate genes in pathways of interest: *CRAT, F3, GCR, ICAM, IFNγ, IL6, iNOS, OGG1, TLR2*	Yes (proportion of lymphocytes and neutrophils)
Lange et al. [26]	2012	Leukocytes	PCR and Pyrosequencing	Alu and LINE-1 elements: coverage of 15,000 different Alu elements/genome.	Yes (proportion of lymphocytes)
Wielscher et al. [24]	2015	Serum (Cell-Free DNA)	MSRE Enrichment and qPCR	63 candidate loci (markers from a 450 K DNAm of lung tissue from those with COPD)	N/A (cell-free DNA)

*Abbreviations*: *PCR* Polymerase chain reaction, *qPCR* Quantitative polymerase chain reaction, *MSRE* Methyl sensitive restriction enzyme

The two studies with a candidate-loci approach performed either bisulfite conversion, followed by polymerase chain reaction (PCR) and pyrosequencing [25], or methylation-sensitive restriction enzymes (MSRE) and quantitative polymerase chain reaction (qPCR) [24]. Bisulfite conversion followed by PCR and pyrosequencing was used to measure Alu and LINE-1 methylation [26].

### EWAS quality control

All three EWAS [21–23] used different quality control measures. These involved the use of duplicate samples, sex prediction, the removal of low-quality samples due to inadequate hybridisation, bisulfite conversion and staining signal, and exclusion of probes with a low call rate. Correction for potential batch effects was attempted in all EWAS by including in the regression model batch-related variables, which were either defined a priori [21, 23] or identified through principal component analysis [22]. The use of ComBat or other correction programs for batch effects were not reported.

### Candidate-loci and global methylation quality control

Two studies [25, 26] reported performing bisulfite conversion validation. However, no information regarding samples excluded due to insufficient bisulfite conversion was stated. To ensure primer specificity in qPCR, Wielscher et al. performed melting temperature assessments. Samples were processed in triplicate and then averaged in two studies [25, 26]. Wielscher et al. used a subset of 16 duplicates to assess technical accuracy [24].

### Biological sample

Five studies [21–23, 25, 26] measured DNA methylation in leukocytes and one study [24] assessed DNA methylation in serum (cell-free DNA). Four studies [21, 22, 25, 26] reported adjustment for cell type in the processing of the data. Qiu et al. and Wielscher et al. did not report adjustment for cell type, and Wielscher et al. used cell-free DNA extracted from serum. All other articles adjusting for differing white cells used relative proportions of the cell types, which were directly measured.

### Association of DNA methylation and COPD

All three studies assessing DNA methylation with COPD status reported associations.

Qiu et al. reported that COPD was associated with 3565 differentially methylated sites in the ICGN discovery cohort (FDR-corrected *p*-value < 0.05) when analyses were conducted without adjustment for confounders (age, sex, smoking status, pack-years, and batch effects). Of note, CpG site cg02181506 in the *SERPINA1* gene on chromosome 14 was top ranked in unadjusted analyses (hypomethylation associated with COPD) (*p*-value = 7.3 × 10^−22^), second ranked in the adjusted analysis (FDR-corrected *p*-value = 3.4 × 10^−10^), and replicated in the EOCOPD cohort (*p*-value = 5.1 × 10^−4^) in the unadjusted analysis. Between both cohorts there were 349 CpG sites associated with all three phenotypes: COPD, FEV1 and FEV1/FVC values (FDR-corrected *p*-value < 0.05), one of these being another *SERPINA1* CpG site (cg24621042).

Wielscher et al. reported four significant (FDR-corrected *p*-value < 0.05) CpG sites - cg05979020 (*HOXD10*), cg05964935 (N/A), cg05769349 (*TBX5*) and cg10384245 (*ADCYAP1*) - from their COPD case-control comparison. All four were hypermethylated in the presence of COPD and none were included in any published tabulations of results from the Qiu et al. report.

Lange et al. reported that hypermethylation of Alu elements was significantly associated (unadjusted *p*-value = 0.046) with a lower odds ratio of COPD (OR 0.80: 0.64–0.99). No significant association with LINE-1 methylation was observed.

### Association of DNA methylation and lung function

Four of the five [22, 23, 25, 26] studies assessing the association of lung function with DNA methylation reported significant findings.

Marioni et al. in an EWAS of FEV1 adjusted for age, sex, height, and smoking, based on the Infinium HumanMethylation450 BeadChip, in 1092 older adults found no probes passed the Bonferroni significance threshold (1.1 × 10^−7^), but 2 sites reached *p* < 1 × 10^−5^ (Chromosome 2, cg14961391, origin recognition complex, subunit 4; Chromosome 14, cg23710823, POTE ankyrin domain family, member M). None were included in any published tabulations of results from Qiu et al. or Bell et al.

Qiu et al. reported significant associations of lung function measures (FEV1 and FEV1/FVC), publishing effect estimates for the top 100 (by *p*-value) which were associated with both measures (e.g. *FXYD1*-cg27461196; *SERPINA1*-cg02181506). All, except one, of the top CpG sites (cg21969640) from Qiu et al. were also present in the more recent Infinium HumanMethylation450 BeadChip, which was used by Marioni et al. but were not found to be significant in this other study.

Bell at al. identified one significant (Bonferroni corrected *p*-value  < 0.05) CpG site associated with FEV1-cg16463460 in the *WT1* gene (*p* = 5.31 × 10^−7^; Beta-value = −0.035). This CpG site, which was present in both Infinium HumanMethylation BeadChips, was not reported as significant in the other included studies.

Lepeule et al. examining associations of methylation in the promoter region of nine genes, involved in inflammation and oxidative stress, with FEV1, FVC, FEV1/FVC and maximum mid-expiratory flow (MMEF) observed decreased methylation in *CRAT, F3, iNOS, OGG1* and *TLR2* to be associated with worse lung function. They also reported that some of these associations (for example, that of FEV1 with methylation of *TLR2*) may alter with age. Hypomethylation of *IFNγ* and *IL6* was associated with better lung function. Although Lepeule et al. had lung function and methylation data at two time points (388 men, average 4 years 7 months apart) authors reported changes in lung function were too small to consider examining longitudinal change in lung function with longitudinal change in methylation. The sites tested by Lepeule et al. could not be compared to those tested in the other studies.

## Discussion

To our knowledge, this is the only systematic review examining associations of lung function, or COPD, with DNA methylation profiles in peripheral blood. This review assessed 1115 unique articles, subsequently including six. DNA methylation profiles in those with reduced lung function or COPD may differ to that of a person with normal healthy lung function, but we found no consistent findings within the published data. While our manuscript was under review, Busch et al. published findings from a small sample (*n* = 362) of African-American smokers from the PA-SCOPE study, who were recruited during inpatient hospitalisation for acute exacerbation of COPD [29]. One of their 12 hits (FDR < 10%) for COPD was reported by Qiu et al. in the ICGN cohort (*FXYD1*-cg27461196) with an FDR-corrected *p*-value of 0.08 and none of the others had been reported previously.

The lack of consistency within the published data could be related to the considerable heterogeneity across the studies in several aspects of study design, laboratory methods and statistical analyses. All studies performed cross-sectional analysis and reported associations that are subject to reverse causality. COPD or reduced lung function could be the cause of the altered DNA methylation profiles, rather than the result. None of the studies provided information on whether the samples had been collected during an exacerbation episode or not (except for Busch et al., whose participants were hospitalised for a COPD exacerbation). However, most were population-based studies with collection of samples either at baseline or at follow-up for all participants, making it unlikely that samples were collected during an exacerbation episode. Wielscher et al. who used COPD patient samples collected from a medical university may be an exception, but we could not check it further. The adjustment for confounders varied considerably across studies, and although most adjusted for age and smoking, the way the latter was treated varied too. Three studies reported and adjusted for the proportions of smokers and pack-years [23, 25, 26], one study did not provide information on smoking history, but adjusted for smoking status [21]. However, two studies neither provided data on smoking history nor adjusted for smoking [22, 24]. The different way in dealing with smoking history may have contributed to the different findings across the studies. While some studies reported the proportions of different races/ethnicities in their sample [21, 23, 25, 26] and adjusted for these in their analyses [25, 26], others did not even report it [22, 24]. This raises the question of whether the findings of some of the studies may be affected by genetic and non-genetic factors specific to the different races/ethnicities, which are known to influence lung function.

There is currently no standardised, or optimal, procedure for the measurement of DNA methylation, and although apparently rigorous, laboratory quality control for both EWAS [21–23] and candidate studies varied. There were different strategies to account for laboratory methods and to account for cell distribution. Qiu et al. did not adjust for white cell counts despite reporting DNA methylation of leukocytes as a whole, and this omission could lead to confounding by the methylation profiles from each cell type [30]. Such adjustment may be particularly important in diseases which have a characteristic immune response, such as the neutrophilic inflammation seen in COPD [31], or common environmental exposures (e.g. tobacco smoke) that have also been shown to be associated with changes in blood cell counts. All three EWAS corrected for potential batch effects.

Notably, there were also differences in the statistical approach to adjustment for potential confounders. This is important as epigenome association studies are susceptible to confounding (i.e. observed associations with one outcome may be due to true associations with another factor), and may be of particular relevance to COPD, which is strongly related to smoking and is associated with multiple morbidities. While the association of an outcome with DNA methylation can be confounded by several exposures, DNA methylation may be the result of exposures that are on the causal pathway to disease (i.e. exposures not acting as confounders) and a careful approach to adjustment for such exposures is needed - this should depend on the underlying scientific question that is being considered.

No power calculations for identification of epigenome wide associations were reported, but attempts were made by many to adjust for multiple testing. Lepeule et al. (using a candidate-loci approach) failed to correct for multiple testing. Except for Qiu et al., who attempted to replicate their findings from ICGN within EOCOPD, all other reports were based on single studies.

This systematic review was carried out with a predefined protocol registered with PROSPERO and in accordance with the PRISMA checklist. Articles were identified using three large online databases using a comprehensive selection of search terms (consisting of both MeSH and free text) with additional simplified searches to identify grey literature. To make the searches as sensitive as possible, no limits were imposed on the search regarding date of publication, language, or article type.

Screening of articles, full-text review and data extraction was carried out by two investigators independently, with each providing reasons for exclusion for each article (see Additional files 2: Table S2). Limitations of this review were that: 1) conference abstracts, which may have contained potentially relevant emerging data, were excluded. However, these usually do not reflect the final conclusion of the study, often require corrections prior to publication and the amount of information on the methods is limited; 2) abstracts of articles had to be available in English; and 3) we only searched online databases from the USA and Europe.

## Conclusion

There were no consistent findings across the identified studies examining the association of lung function or COPD with peripheral blood DNA methylation, possibly due to the heterogeneity of methods used, and even with access to full sets of unpublished results we would hesitate to combine results from these studies because of the different laboratory quantification, quality control and analytical methods used. Reports were based on cross-sectional analyses of DNA methylation and lung function/COPD (even though participants were taking part in cohort studies) and methylation patterns could be the cause of, or a result of, altered lung function. Some reported associations might have been related to smoking rather than to disease or lung function *per se*. Furthermore, COPD patients may also suffer from other conditions, or take drug therapies, which may be associated with differential DNA methylation.

Future longitudinal studies, with serial measurements of DNA methylation at yearly intervals, are required to assess the temporal sequence of disease onset and peripheral blood methylation. Harmonization of methods, and established guidelines for quality control and processing of DNA methylation data would facilitate meta-analysis of epigenome association studies. There is some evidence of associations with highly biologically plausible areas (e.g. *SERPINA1*), and targeted analyses to investigate these further are warranted.

## Additional files

Additional file 1: Tables S1. Search terms (*n* = 92) used within MEDLINE, EMBASE and Web of Science. (DOCX 13 kb)
Additional file 2: Tables S2. First 25 exclusion categorisations for both investigators. This process was repeated for all 1155 articles undergoing Title and Abstract screening. (DOCX 17 kb)

## Abbreviations

AAT: Alpha-1 antitrypsin
CGI: CpG island
COPD: Chronic obstructive pulmonary disease
CpG: 5’-cytosine-phosphate-guanine-3’ sequence
DNA: Deoxyribonucleic acid
DZ: Dizygotic
EOCOPD: Boston Early Onset COPD Study
EWAS: Epigenome-wide association study
FDR: False discovery rate
FEV1: Forced expiratory volume in one second
FVC: Forced vital capacity
GOLD: Global Initiative for Chronic Obstructive Lung Disease
ICGN: International COPD Genetics Network
LINE-1: Long interspersed nuclear element 1
MeSH: Medical Subject Headings
MMEF: Maximum mid-expiratory flow
MSRE: Methyl sensitive restriction enzyme
MZ: Monozygotic
N/A: Not available/not applicable
PA-SCOPE: Pennsylvania Study of Chronic Obstructive Pulmonary Exacerbations
PCR: Polymerase chain reaction
PRISMA: Preferred Reporting Items for Systematic Reviews and Meta-Analyses
qPCR: Quantitative polymerase chain reaction
RNA: Ribonucleic acid
WoS: Web of Science

## References

[1] Smith ZD, Meissner A (2013). DNA methylation: roles in mammalian development. Nat Rev Genet.

[2] Li E, Beard C, Jaenisch R (1993). Role for DNA methylation in genomic imprinting. Nature.

[3] Ng H-H, Adrian B (1999). DNA methylation and chromatin modification. Curr Opin Genet Dev.

[4] Bird AP (1980). DNA methylation and the frequency of CpG in animal DNA. Nucleic Acids Res.

[5] Jones PA (2012). Functions of DNA methylation: islands, start sites, gene bodies and beyond. Nat Rev Genet.

[6] Bird AP (1985). CpG-rich islands and the function of DNA methylation. Nature.

[7] Adeloye D, Chua S, Lee C, Basquill C, Papana A, Theodoratou E, Nair H, Gasevic D, Sridhar D, Campbell H (2015). Global and regional estimates of COPD prevalence: systematic review and meta-analysis. J Glob Health.

[8] Halbert R, Natoli J, Gano A, Badamgarav E, Buist AS, Mannino DM (2006). Global burden of COPD: systematic review and meta-analysis. Eur Respir J.

[9] Kotaniemi JT, Sovijärvi A, Lundbäck B. Chronic obstructive pulmonary disease in Finland: Prevalence and risk factors, COPD: J Chron Obstruct Pulmon Dis. 2005;2:331–9. 10.1080/15412550500218122 17146998

[10] Løkke A, Lange P, Scharling H, Fabricius P, Vestbo J (2006). Developing COPD: a 25 year follow up study of the general population. Thorax.

[11] Mannino DM, Buist AS (2007). Global burden of COPD: risk factors, prevalence, and future trends. Lancet.

[12] Lindberg A, Bjerg-Bäcklund A, Rönmark E, Larsson L-G, Lundbäck B (2006). Prevalence and underdiagnosis of COPD by disease severity and the attributable fraction of smoking: report from the Obstructive Lung Disease in Northern Sweden Studies. Respir Med.

[13] Shahab L, Jarvis M, Britton J, West R (2006). Prevalence, diagnosis and relation to tobacco dependence of chronic obstructive pulmonary disease in a nationally representative population sample. Thorax.

[14] Amaral AF, Coton S, Kato B, Tan WC, Studnicka M, Janson C, Gislason T, Mannino D, Bateman ED, Buist S (2015). Tuberculosis associates with both airflow obstruction and low lung function: BOLD results. Eur Respir J.

[15] Hooper R, Burney P, Vollmer WM, Mcburnie MA, Gislason T, Tan WC, Jithoo A, Kocabas A, Welte T, Buist AS (2012). Risk factors for COPD spirometrically defined from the lower limit of normal in the BOLD project. Eur Respir J.

[16] Gao X, Jia M, Zhang Y, Breitling LP, Brenner H (2015). DNA methylation changes of whole blood cells in response to active smoking exposure in adults: a systematic review of DNA methylation studies. Clin Epigenetics.

[17] Zhang Y, Yang R, Burwinkel B, Breitling LP, Brenner H (2014). F2RL3 methylation as a biomarker of current and lifetime smoking exposures. Environ Health Perspect.

[18] Moher D, Liberati A, Tetzlaff J, Altman DG (2009). Preferred reporting items for systematic reviews and meta-analyses: the PRISMA statement. Ann Intern Med.

[19] Wilson DO, Weissfeld JL, Balkan A, Schragin JG, Fuhrman CR, Fisher SN, Wilson J, Leader JK, Siegfried JM, Shapiro SD (2008). Association of radiographic emphysema and airflow obstruction with lung cancer. Am J Respir Crit Care Med.

[20] Wang L, Aakre JA, Jiang R, Marks RS, Wu Y, Chen J, Thibodeau SN, Pankratz VS, Yang P (2010). Methylation markers for small cell lung cancer in peripheral blood leukocyte DNA. J Thorac Oncol.

[21] Marioni RE, Shah S, Mcrae AF, Ritchie SJ, Muniz-Terrera G, Harris SE, Gibson J, Redmond P, Cox SR, Pattie A (2015). The epigenetic clock is correlated with physical and cognitive fitness in the Lothian Birth Cohort 1936. Int J Epidemiol.

[22] Bell JT, Tsai P-C, Yang T-P, Pidsley R, Nisbet J, Glass D, Mangino M, Zhai G, Zhang F, Valdes A (2012). Epigenome-wide scans identify differentially methylated regions for age and age-related phenotypes in a healthy ageing population. Plos Genet.

[23] Qiu W, Baccarelli A, Carey VJ, Boutaoui N, Bacherman H, Klanderman B, Rennard S, Agusti A, Anderson W, Lomas DA, Demeo DL (2012). Variable DNA methylation is associated with chronic obstructive pulmonary disease and lung function. Am J Respir Crit Care Med.

[24] Wielscher M, Vierlinger K, Kegler U, Ziesche R, Gsur A, Weinhäusel A (2015). Diagnostic performance of plasma DNA methylation profiles in lung cancer, pulmonary fibrosis and COPD. EBioMedicine.

[25] Lepeule J, Baccarelli A, Tarantini L, Motta V, Cantone L, Litonjua AA, Sparrow D, Vokonas PS, Schwartz J (2012). Gene promoter methylation is associated with lung function in the elderly: the Normative Aging Study. Epigenetics.

[26] Lange NE, Sordillo J, Tarantini L, Bollati V, Sparrow D, Vokonas P, Zanobetti A, Schwartz J, Baccarelli A, Litonjua AA (2012). Alu and LINE-1 methylation and lung function in the normative ageing study. BMJ Open.

[27] Deary IJ, Gow AJ, Pattie A, Starr JM (1921). Cohort profile: the Lothian Birth Cohorts of 1921 and 1936. Int J Epidemiol.

[28] Moayyeri A, Hammond CJ, Valdes AM, Spector TD (2013). Cohort profile: TwinsUK and healthy ageing twin study. Int J Epidemiol.

[29] Busch R, Qiu W, Lasky-Su J, Morrow J, Criner G, Demeo D (2016). Differential DNA methylation marks and gene comethylation of COPD in African-Americans with COPD exacerbations. Respir Res.

[30] Houseman EA, Kim S, Kelsey KT, Wiencke JK (2015). DNA methylation in whole blood: uses and challenges. Curr Environ Health Rep.

[31] O’Donnell R, Breen D, Wilson S, Djukanovic R (2006). Inflammatory cells in the airways in COPD. Thorax.

[32] Silverman EK, Chapman HA, Drazen JM, Weiss ST, Rosner B, Campbell EJ, O’Donnell WJ, Reilly JJ, Ginns L, Mentzer S (1998). Genetic epidemiology of severe, early-onset chronic obstructive pulmonary disease. Risk to relatives for airflow obstruction and chronic bronchitis. Am J Respir Crit Care Med.

