# Supplementary material for: Systematic review of lung function and COPD with peripheral blood DNA methylation in population based studies
Source: BMC Pulm Med. 2017 Mar 20;17:54. doi: 10.1186/s12890-017-0397-3 (PMC5360084; doi:10.1186/s12890-017-0397-3)
Supplement: Additional file 1: Tables S1. — Search terms (n = 92) used within MEDLINE, EMBASE and Web of Science. (DOCX 13 kb) [file 12890_2017_397_MOESM1_ESM.docx]

**Table S1.** Search terms (n=92) used within MEDLINE, EMBASE and Web of Science.

| **Subject Heading** | **Search Terms Used** |
| --- | --- |
| **Epigenetics**  **DNA methylation**  **Alu and LINE-1** | (DNA Methylation* or Methylat* or Hypomethylat* or Hypermethylat* or Methylation*, DNA or Gene silencing or Imprinting or Epigenetic* or Epigenomic* or Epigenesis, genetic or Methyl transferase* or Long Interspersed Nucleotide Element* or LINE Repeat Sequence* or Repeat Sequence*, LINE or Sequence*, LINE Repeat or Long Interspersed DNA Sequence Element* or LINE-1 Element* or Element*, LINE-1 or LINE-1 Element* or Element*, L1 or L1 Element* or Element*, Jockey or Jockey Element* or Alu Element* or Element*, Alu or Alu Famil* or Famil*, Alu or Alu Repetitive Sequence* or Repetitive Sequence*, Alu or Sequence*, Alu Repetitive or Alu-Like Repetitive Sequence*). |
| **AND** | |
| **Chronic obstructive pulmonary disease**  **Lung function**  **Cigarette smoking** | (COPD or Chronic Obstructive Pulmonary Disease* or COAD or Chronic Obstructive Airway Disease* or Chronic Obstructive Lung Disease* or Airflow Obstruction*, Chronic or Chronic Airflow Obstruction* or Small Airway Disease or Disease of the small airways or Chronic Bronchitis or Pulmonary Emphysema* or Emphysema*, Pulmonary or Focal Emphysema* or Emphysema*, Focal or Panacinar Emphysema* or Emphysema*, Panacinar or Panlobular Emphysema* or Emphysema*, Panlobular or Centriacinar Emphysema* or Emphysema*, Centriacinar or Centrilobular Emphysema* or Emphysema*, Centrilobular or Airway Obstruction* or Lung disease*, obstructive or Obstructive lung disease* or Obstructive pulmonary disease* or Pulmonary disease*, obstructive or Lung Function or LFT* or Forced expiratory volume in one second or FEV1 or Forced expiratory volume in 1 second or Forced vital capacit* or FVC or Forced expiratory volume percent predicted or FEV1 FVC or FEV1 FVC or FEV1 FVC ratio or FEV1 FVC ratio or FEV1% or Function Test*, Respiratory or Respiratory Function Test* or Test*, Respiratory Function or Pulmonary Function Test* or Function Test*, Pulmonary or Test*, Pulmonary Function or Lung Function Test* or Function Test*, Lung or Test*, Lung Function or Spirometr* or Tobacco Smoke Exposure* or Cigarette smok* or Tobacco smok* or Cigarette* or Cigarette smoking or Cigar Smok* or Smoking, Cigar or Smoking, Tobacco or Pipe Smok* or Smoking, Pipe or Smoking, Cigarette) |
