# Supplementary material for: Systematic review of lung function and COPD with peripheral blood DNA methylation in population based studies
Source: BMC Pulm Med. 2017 Mar 20;17:54. doi: 10.1186/s12890-017-0397-3 (PMC5360084; doi:10.1186/s12890-017-0397-3)
Supplement: Additional file 2: Tables S2. — First 25 exclusion categorisations for both investigators. This process was repeated for all 1155 articles undergoing Title and Abstract screening. (DOCX 17 kb) [file 12890_2017_397_MOESM2_ESM.docx]

**Table S2.** First 25 exclusion categorisations for both investigators. This process was repeated for all 1,155 articles undergoing Title and Abstract screening.

| **Year** | **Author** | **Title** | **Reason for Exclusion DJ** | **Reason for Exclusion MM** |
| --- | --- | --- | --- | --- |
| 1982 | Brown et al. | A novel double-isotope technique for the enzymatic assay of plasma histamine: application to estimation of mast cell activation assessed by antigen challenge in asthmatics | No measure of DNA methylation DJ | No measure of DNA methylation MM |
| 1993 | Kawakami et al. | A novel fucosylated glycosphingolipid with a Gal beta 1-4Glc beta 1-3Gal sequence in plerocercoids of the parasite, Spirometra erinacei | in vivo/in vitro (Not in Humans) DJ | Population not defined by COPD or LFT MM |
| 2001 | Rioux et al. | 4-(methylnitrosamino)-1-(3-pyridyl)-1-butanone modulation of cytokine release in U937 human macrophages | Population not defined by COPD or LFT DJ | Population not defined by COPD or LFT MM |
| 2001 | Zochbauer-Muller et al. | 5' CpG island methylation of the FHIT gene is correlated with loss of gene expression in lung and breast cancer | Population not defined by COPD or LFT DJ | Population not defined by COPD or LFT MM |
| 2005 | Niot-Mansart et al. | A competitive ELISA detecting 7-methylguanosine adduct induced by N-nitrosodimethylamine exposure | Population not defined by COPD or LFT DJ | Population not defined by COPD or LFT MM |
| 2009 | Biggen et al. | A Panel Study on Epigenetics, Markers of Oxidative Stress, and Lung Function Among Children with Respiratory Disease Exposed to Industrial Air Pollution | Articles not original research/ not full text published in peer-reviewed journal DJ | Articles not original research/ not full text published in peer-reviewed journal MM |
| 2010 | Richards et al. | A role for statin therapy in Alzheimer's disease | Population not defined by COPD or LFT DJ | Population not defined by COPD or LFT MM |
| 2011 | Anonymous et al. | A Model for molecular progression to squamous carcinoma | Articles not original research/ not full text published in peer-reviewed journal DJ | Population not defined by COPD or LFT MM |
| 2011 | Caliebe et al. | A familial disorder with genome-wide disturbance of DNA methylation at both paternally and maternally imprinted loci | Population not defined by COPD or LFT DJ | Population not defined by COPD or LFT MM |
| 2011 | De Castro et al. | A phase i study of the combination of decitabine with lenalidomide for patients with high risk (IPSS Int-2 or high) myelodysplastic syndrome | Population not defined by COPD or LFT DJ | Population not defined by COPD or LFT MM |
| 2011 | Field et al. | A pilot lung cancer early detection study in a primary care practice in knowsley, merseyside: The liverpool lung project primary care implementation programme (llppcip) | No measure of DNA methylation DJ | Population not defined by COPD or LFT MM |
| 2011 | Rao et al. | 3-Deazaneplanocin A (DZNep) inhibits repression of tumor suppressor genes and abrogates tumorigenicity of lung cancer cells mediated by cigarette smoke | Population not defined by COPD or LFT DJ | Population not defined by COPD or LFT MM |
| 2012 | Accomando et al. | A novel method for the detection of circulating NK cells in archived blood reveals a decrease in NK cells in head and neck squamous cell carcinoma | Population not defined by COPD or LFT DJ | Population not defined by COPD or LFT MM |
| 2012 | Joubert et al. | 450K epigenome-wide scan identifies differential DNA methylation in newborns related to maternal smoking during pregnancy | Population not defined by COPD or LFT DJ | Population not defined by COPD or LFT MM |
| 2012 | Qiu et al. | A Chronic Obstructive Pulmonary Disease-Variable DNA Methylation Is Associated with Chronic Obstructive Pulmonary Disease and Lung Function | Articles not original research/ not full text published in peer-reviewed journal DJ | Articles not original research/ not full text published in peer-reviewed journal MM |
| 2012 | Wang et al. | 4-(Methylnitrosamino)-1-(3-pyridyl)-1-butanone induces retinoic acid receptor beta hypermethylation through DNA methyltransferase 1 accumulation in esophageal squamous epithelial cells | Population not defined by COPD or LFT DJ | Population not defined by COPD or LFT MM |
| 2012 | Wu et al. | A prospective study of arsenic exposure, arsenic methylation capacity, and risk of cardiovascular disease in Bangladesh | Population not defined by COPD or LFT DJ | Population not defined by COPD or LFT MM |
| 2013 | Anonymous | 2nd National Congress on Medicinal Plants | Population not defined by COPD or LFT DJ | Population not defined by COPD or LFT DJ |
| 2013 | Nishihara et al. | A prospective study of duration of smoking cessation and colorectal cancer risk by epigenetics-related tumor classification | Population not defined by COPD or LFT DJ | Clinical sample not peripheral blood (i.e. lung tissue) MM |
| 2014 | Barrett et al. | A Smoking Gun? Epigenetic Markers of Tobacco Use History | Population not defined by COPD or LFT DJ | Articles not original research/ not full text published in peer-reviewed journal MM |
| 2014 | Guida et al. | 0220 Identification of short-term, long-term and lifelong DNA methylation markers of exposure to tobacco smoke: evidence from EPIC and NOWAC studies | Articles not original research/ not full text published in peer-reviewed journal DJ | Population not defined by COPD or LFT (Smoke Exposure) MM |
| 2014 | Yu et al. | A meta-analysis of lifestyle factors with MPO and GSTM1 human genes in lung cancer prevention | Population not defined by COPD or LFT DJ | Population not defined by COPD or LFT MM |
| 2015 | Li et al. | A potential synergy between incomplete arsenic methylation capacity and demographic characteristics on the risk of hypertension: findings from a cross-sectional study in an arsenic-endemic area of inner Mongolia, China | Population not defined by COPD or LFT DJ | Population not defined by COPD or LFT MM |
| 2015 | Philibert et al. | A quantitative epigenetic approach for the assessment of cigarette consumption | Population not defined by COPD or LFT (Smoke Exposure) DJ | Population not defined by COPD or LFT (Smoke Exposure) MM |
| 2015 | Philibert et al. | A next-gen approach to the assessment and treatment of heavy alcohol consumption | Articles not original research/ not full text published in peer-reviewed journal DJ | Population not defined by COPD or LFT MM |
